# Metformin use correlated with lower risk of cardiometabolic diseases and related mortality among US cancer survivors: evidence from a nationally representative cohort study

**DOI:** 10.1186/s12916-024-03484-y

**Published:** 2024-06-26

**Authors:** Yukun Li, Xiaoying Liu, Wenhe Lv, Xuesi Wang, Zhuohang Du, Xinmeng Liu, Fanchao Meng, Shuqi Jin, Songnan Wen, Rong Bai, Nian Liu, Ribo Tang

**Affiliations:** 1 Department of Cardiology, Beijing Anzhen Hospital, Capital Medical University, Beijing, 100012 China; 2 National Clinical Research Center for Cardiovascular Diseases, Beijing, 100012, China; 3 Banner University Medical Center Phoenix, College of Medicine University of Arizona, Phoenix, AZ 85123 USA; 4 Department of Cardiovascular Medicine, Mayo Clinic, Scottsdale, AZ 85259 USA

**Keywords:** Cardiometabolic disease, Cancer, Cardio-oncology, Metformin, Oxidative stress

## Abstract

**Background:**

In the USA, the prolonged effective survival of cancer population has brought significant attention to the rising risk of cardiometabolic morbidity and mortality in this population. This heightened risk underscores the urgent need for research into effective pharmacological interventions for cancer survivors. Notably, metformin, a well-known metabolic regulator with pleiotropic effects, has shown protective effects against cardiometabolic disorders in diabetic individuals. Despite these promising indications, evidence supporting its efficacy in improving cardiometabolic outcomes in cancer survivors remains scarce.

**Methods:**

A prospective cohort was established using a nationally representative sample of cancer survivors enrolled in the US National Health and Nutrition Examination Survey (NHANES), spanning 2003 to 2018. Outcomes were derived from patient interviews, physical examinations, and public-access linked mortality archives up to 2019. The Oxidative Balance Score was utilized to assess participants’ levels of oxidative stress. To evaluate the correlations between metformin use and the risk of cardiometabolic diseases and related mortality, survival analysis of cardiometabolic mortality was performed by Cox proportional hazards model, and cross-sectional analysis of cardiometabolic diseases outcomes was performed using logistic regression models. Interaction analyses were conducted to explore the specific pharmacological mechanism of metformin.

**Results:**

Among 3995 cancer survivors (weighted population, 21,671,061, weighted mean [SE] age, 62.62 [0.33] years; 2119 [53.04%] females; 2727 [68.26%] Non-Hispanic White individuals), 448 reported metformin usage. During the follow-up period of up to 17 years (median, 6.42 years), there were 1233 recorded deaths, including 481 deaths from cardiometabolic causes. Multivariable models indicated that metformin use was associated with a lower risk of all-cause (hazard ratio [HR], 0.62; 95% confidence interval [CI], 0.47–0.81) and cardiometabolic (HR, 0.65; 95% CI, 0.44–0.97) mortality compared with metformin nonusers. Metformin use was also correlated with a lower risk of total cardiovascular disease (odds ratio [OR], 0.41; 95% CI, 0.28–0.59), stroke (OR, 0.44; 95% CI, 0.26–0.74), hypertension (OR, 0.27; 95% CI, 0.14–0.52), and coronary heart disease (OR, 0.41; 95% CI, 0.21–0.78). The observed inverse associations were consistent across subgroup analyses in four specific cancer populations identified as cardiometabolic high-risk groups. Interaction analyses suggested that metformin use as compared to non-use may counter-balance oxidative stress.

**Conclusions:**

In this cohort study involving a nationally representative population of US cancer survivors, metformin use was significantly correlated with a lower risk of cardiometabolic diseases, all-cause mortality, and cardiometabolic mortality.

**Supplementary Information:**

The online version contains supplementary material available at 10.1186/s12916-024-03484-y.

## Key points

Question:

Is metformin use associated with diminished risk of cardiometabolic diseases and related mortality in cancer survivors? If so, what mechanisms contribute to this inverse association with cardiometabolic risk?

Findings:

In this cohort study of 3995 cancer survivors over a median period of 6.42 years, metformin use was correlated with decreased risks of cardiometabolic diseases, all-cause and cardiometabolic mortality, likely due to its oxidative stress antagonism.

Meaning:

These study findings indicated that metformin use was associated with improved cardiometabolic health, leading to enhanced overall survival and quality of life in cancer survivors. Moreover, targeting oxidative stress may be crucial in developing cardiometabolic protective drugs for patients with cancer in the future.

**Supplementary Information:**

The online version contains supplementary material available at 10.1186/s12916-024-03484-y.

## Background

Cardiometabolic disease (CMD) and cancer are two major global public health concerns [1, 2]. Recent advancements in cancer therapies including chemotherapy, radiotherapy, targeted therapy, and immunotherapy, have led to an expanding population of cancer survivors (CS). Two-thirds of patients diagnosed with cancer survive beyond 5 years post-diagnosis. However, the extended lifespan of CS presents new challenges for long-term care and comorbidity management. CMD has emerged as the primary comorbidity in patients with cancer, ranking as the leading cause of noncancer deaths in the CS population [3–5]. This increased risk of CMD and related mortality arose from various factors, including direct effects of cancers, anticancer treatments (including radiation and chemotherapy), pre-existing cardiometabolic risk factors (such as dysglycemia, dyslipidemia, and obesity), and physical deconditioning [3].

Apart from the previously mentioned risk factors, cancer is hypothesized as a type of metabolic disease. The link between cancer and CMD can possibly be explained by the abnormal metabolic phenotype of cancer cells (known as the Warburg effect) and elevated levels of oxidative stress [6, 7], particularly considering that the cardiovascular system is highly energy-consuming and sensitive to altered metabolic patterns. Currently, there are no specific guidelines for managing and preventing cardiometabolic complications in this highly metabolically challenged group, relying instead on recommendations extrapolated from general populations. In this context, urgent attention is required for the development of prevention strategies aimed at alleviating the CMD burden in cancer survivors.

Both experimental and clinical data indicated that metformin, the primary oral antihyperglycemic agent with pharmacological adenosine 5’ monophosphate-activated protein kinase (AMPK) activation, could improve the cardiometabolic status in populations with obesity, diabetes, or psychiatric disorders [8]. This improvement was attributed to mechanisms including oxidative stress inhibition and redox rebalance [9–11]. To date, scarce studies have investigated the correlations of metformin use with CMD risk and CMD-related mortality in patients with cancer. Therefore, this study aimed to investigate the correlations between metformin use and the risk of CMD / CMD-related mortality, as well as the role of the antioxidative stress mechanism in a nationally representative sample of US cancer survivors with sufficient follow-up time. Our study findings offered crucial insights into the clinical application, mechanistic understanding, and future development of effective interventions to mitigate the increasing trend of cardiometabolic dysfunction among cancer survivors.

## Methods

### Study sample and population

This cohort study utilized a nationally representative population of cancer survivors from the National Health and Nutrition Examination Survey (NHANES) [12, 13]. This study’s analysis adhered to the analytical guidelines of NHANES, which adopted a multi-stage stratified systematic sampling design. The sampling and testing processes in NHANES have been thoroughly documented in previously published articles. Briefly, the survey conducted health-related interviews and examinations, encompassing participants from diverse geographical locations and racial/ethnic backgrounds to ensure its nationwide representativeness. The NHANES protocols received approval from the National Center for Health Statistics research ethics review board. Written informed consent was obtained from all participants involved in the survey.

NHANES is an ongoing series of cross-sectional studies conducted in 2-year cycles. Except for mortality data, which was obtained from the 2019 Public-Use Linked Mortality Files, all other participant data, including the exposure of interest (metformin use) and covariates of our study, were collected during the survey cycle in which the participants were enrolled. Specifically, NHANES gathered health-related information through a combination of health interviews, medical measurements, and laboratory assessments. One or more individuals per household were selected to participate. Data was collected from each participant via face-to-face interviews conducted in the respondents’ homes. Medical measurements were performed in specially equipped mobile centers that travel to various locations throughout the country. Subsequently, participants were invited to provide biological samples and undergo laboratory assessments. In the Cox proportional hazards regression analysis, follow-up was calculated using person-months from the date of the interview to either the date of death or the deadline of the 2019 Public-Use Linked Mortality Files (December 31, 2019). Comprehensive methodology and protocols can be found on the NHANES website.

**Fig. 1:**
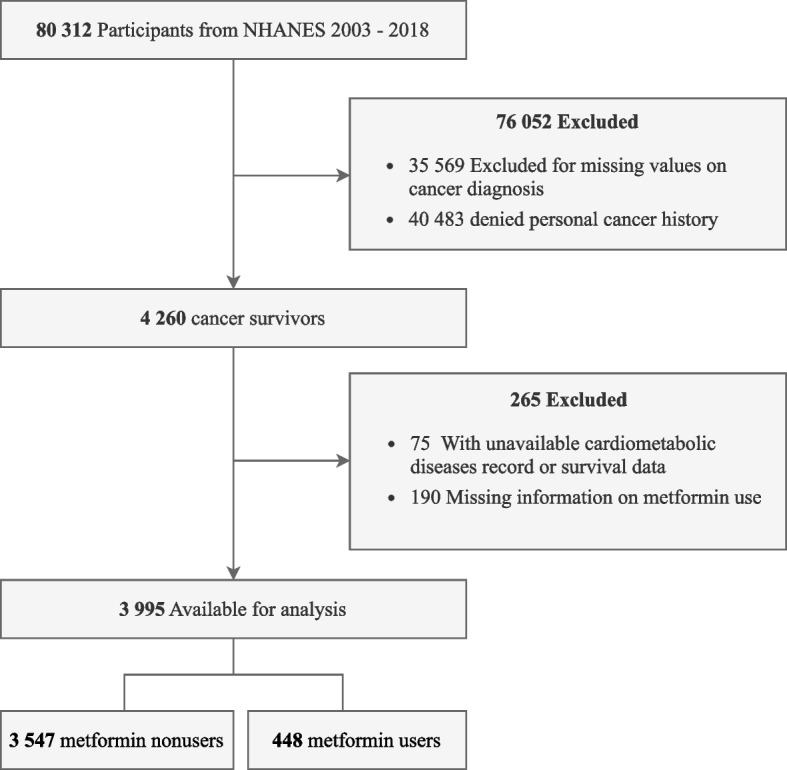
Flowchart diagram of the screening and enrollment of study participants

In this study, we analyzed data of sociodemographic variables, lifestyle factors, and medical and medication history from adult cancer survivors with follow-up records spanning eight cycles of NHANES from 2003 to 2018. Cancer diagnosis and type information were obtained through in-person interviews, encompassing details regarding specific cancer type(s), recodes of up to three cancer diagnoses, and the age at each diagnosis. Participants were asked, “Have you ever been told by a doctor or other health professional that you had cancer or a malignancy of any kind?” Those answering “yes” were identified as cancer survivors and further asked “What kind of cancer was it?” and “How old were you when this cancer was first diagnosed?”. The value of variable “years since first cancer diagnosis” was calculated by subtracting the age at the first cancer diagnosis from the participant’s current age. During household interviews, participants were questioned about their prescription medication use in the past month. If their response was affirmative, the participants were further asked to present medication containers for recording. Medication names, upon entry, were systematically aligned with an existing prescription drug database. In cases where medication containers were unavailable, participants were requested to report the medication name verbally. Metformin use was obtained from participants’ self-reports during the in-home questionnaire. Figure 1 illustrates the participant enrollment process in a flowchart.

### Oxidative balance score

This study computed the Overall Oxidative Balance Score (OBS) by totaling the points allocated to each component. A lower OBS score indicated a higher individual oxidative stress level. Sixteen nutrients and four lifestyle-related components were selected for OBS calculation, including fifteen antioxidants and five prooxidants, based on previous studies examining their relationships with oxidative stress (OS) [14, 15].

The scoring allocation scheme for OBS components is detailed in Supplementary Table 1 (Additional file 1: Table S1). For alcohol consumption, the scoring was as follows: nondrinkers received 2 points, nonheavy drinkers (0–15 g/day for women and 0–30 g/day for men) received 1 point, and heavy drinkers (≥ 15 g/day for women and ≥ 30 g/day for men) received 0 points. Notably, smoking exposure was quantified using serum cotinine levels, reflecting both direct tobacco use and environmental tobacco smoke exposure. Other components were categorized into three groups based on gender-specific tertiles. Antioxidants were scored from 0 to 2, progressively assigned from the first to the third tertile. However, prooxidants were scored inversely, with the highest tertile receiving 0 points and the lowest tertile receiving 2 points. Patients with cancer were classified into high OS and low OS groups based on OS level, defined by the lower and higher 50% of OBS values, respectively.

### Outcome definition

In this study, mortality data were obtained from the 2019 public-access linked mortality archives up to December 31, 2019. All-cause and cardiometabolic mortality statuses were acquired from the publicly accessible dataset mentioned above. For participants identified as “deceased,” death cases were coded according to the Tenth Revision of the International Classification of Diseases (ICD-10). The underlying causes of death were categorized into the following 10 types: diseases of the heart; malignant neoplasms; chronic lower respiratory diseases; accidents (unintentional injuries); cerebrovascular diseases; Alzheimer’s disease; diabetes mellitus; influenza and pneumonia; nephritis, nephrotic syndrome, and nephrosis; and all other causes (residual).

Cardiometabolic mortality outcome was defined as the combination of death events resulting from three primary causes: diseases of the heart, cerebrovascular diseases, and diabetes mellitus. Death from accidents (unintentional injuries) was defined as a negative control outcome, which was unlikely to be influenced by the metformin treatment.

Although there is no consensus on the exact scope of CMD, prior studies typically encompassed coronary heart disease, stroke, hypertension, dyslipidemia, and diabetes mellitus within this category. Notably, all metformin users in our study had a history of diabetes comorbidity. Based on previous NHANES-related research on CMD and considering the sample sizes of certain diseases in the NHANES database, along with clinical practice, four common CMDs were included to assess the cardiometabolic diseases risk of cancer survivors: total cardiovascular disease, stroke, hypertension, and coronary heart disease (CHD). Hypertension diagnosis was based on the response “yes” to self-reported hypertension questions (“Have you ever been told by a doctor or other health professional that you had hypertension, also called high blood pressure?”), antihypertension drug usage (“Because of your high blood pressure/hypertension, have you ever been told to take prescribed medicine?”), or abnormal average values in three blood pressure measurements (systolic blood pressure greater than 130 mmHg or diastolic blood pressure greater than 80 mmHg). Stroke was diagnosed among those who responded “yes” to the self-reported stroke question (“Has a doctor or other health professional ever told you that you had a stroke?”). CHD was diagnosed among individuals responding “yes” to the self-reported CHD question (“Has a doctor or other health professional ever told you that you had coronary heart disease?”). The diagnosis of total cardiovascular disease included individuals diagnosed with any or a combination of the following conditions: coronary heart disease, congestive heart failure, heart attack, stroke, or angina.

### Study covariates

We constructed a directed acyclic graph (DAG) to visualize the hypothesized associations of the primary exposure (metformin treatment) with the outcomes of interest (cardiometabolic outcomes of cancer survivors), and potential covariates. The selection of clinical and biochemical covariates incorporated into the DAG was guided by pragmatic considerations and prior mechanistic insights into the pathophysiology of cardiometabolic diseases. The resulting DAG is presented in Supplementary Fig. 1 (Additional file 1: Figure S1).

In our study, covariates including age, gender, ethnicity/race (categorized as non-Hispanic white, non-Hispanic black, Mexican American, other Hispanic, and others), educational level (categorized as less than high school, high school or equivalent, and college or above), and socioeconomic status measured by the poverty to income ratio (PIR = Family income / Poverty threshold for family size and composition) were extracted from interviews and physical examinations. The PIR index was stratified into three levels: < 1.30, 1.30–3.49, and ≥ 3.5. Body mass index (BMI) was calculated as weight (kg) divided by the square of height (m^2^) and categorized into three subgroups based on the cutoff values of 25 and 30, with BMI ≥ 30 indicating obesity. Smoking status was assessed and classified as “never” for individuals who smoked less than 100 cigarettes in their lifetime, “former” for those who had smoked more than 100 cigarettes but currently do not smoke, and “now” for individuals who smoked more than 100 cigarettes in their lifetime and smoke some days or every day. Alcohol consumption status was grouped into five categories: (1) never (< 12 drinks in a lifetime), (2) former (≥ 12 drinks in 1 year and did not drink last year, or did not drink last year but drank ≥ 12 drinks in a lifetime), (3) current mild alcohol use (< two drinks per day for women, < three drinks per day for men), (4) current moderate alcohol use (≥ two drinks per day for women, ≥ three drinks per day for men, or binge drinking ≥ two days per month), and (5) current heavy alcohol use (≥ three drinks per day for women, > four drinks per day for men, or binge drinking on ≥ 5 days per month).

The metabolic equivalent (MET) measured energy metabolism during various daily activities. Physical activity was assessed through the Physical Activity Questionnaire (PAQ) section and quantified as PA(MET-h/week) = MET * weekly frequency * duration of each of physical activity. Distinct MET values were assigned for diverse physical activities by NHANES guidelines, including vigorous work-related activity (MET = 8.0), vigorous leisure-time physical activity (MET = 8.0), moderate work-related activity (MET = 4.0), walking or bicycling for transportation (MET = 4.0), and moderate leisure-time physical activity (MET = 4.0). Participants were categorized into two subgroups based on their PA scores: low-intensity physical activity (PA < 48 MET-h/week) and high-intensity physical activity (PA > 48 MET-h/week). The history of hyperlipidemia, diabetes, and depression (Patient Health Questionnaire-9 [PHQ9] ≥ 10) was identified through questionnaires section. The use of antihypertensive and antihyperlipidemic agents was ascertained based on participants’ self-reported medication usage in the in-home questionnaire. Multiple imputation methods were employed for missing covariate values.

### Statistical analysis

All data analyses incorporated the complex stratified survey design and NHANES sampling weights to ensure national representativeness. Continuous variables in this study were reported as means and standard error of mean (SE), while categorical variables were presented as weighted percentages. Statistical tests were conducted two-sided, and a significance threshold of *p* < 0.05 was applied. Data analysis was conducted from May 1 to August 1, 2023, using R and R Studio (R Foundation for Statistical Computing, Version 4.2.0). Multiple logistic regression models were employed in this study to evaluate the correlation between metformin use and the risk of CMD. Furthermore, multivariable Cox proportional hazards regression models were utilized to assess the impact of metformin use on risks of all-cause mortality and cardiometabolic mortality. The fully adjusted models were adjusted for a range of covariates, including age, gender, race and ethnicity, educational level, PIR, BMI, smoking status, alcohol use, intensity of physical activity, health conditions (including histories of hyperlipidemia, depression, and diabetes), and medication history (antihypertensive agents usage and antihyperlipidemic agents usage) and years since first cancer diagnosis. The final reported outcomes from these analyses were the adjusted odds ratios / hazard ratios and their corresponding 95% confidence intervals (95% CI). The robustness of all logistic regression and Cox proportional-hazards models was further evaluated by calculating the E-value [16], which represents the minimum strength of relationship, on the OR/HR scale, that an unmeasured confounding variable would need to have with both metformin use and cardiometabolic outcomes to entirely suppress the observed correlations, after adjusting for the measured covariates.

To further investigate whether metformin exerted its cardiometabolic protective effect by counteracting oxidative stress, the distinctive nature of interaction analyses was considered in logistic regression and Cox proportional hazards regression models. Notably, Rothman et al. have highlighted that the interaction terms (a*b) in these models only reflected the multiplicative interaction from a statistical perspective, rather than translating into a biologically interpretable additive interaction effect [17]. Accordingly, new variables were created, including four exclusive categories based on combinations of metformin use and oxidative stress levels: (1) metformin users in the low OS group, (2) metformin nonusers in the low OS group, (3) metformin users in the high OS group and (4) metformin nonusers in the high OS group. This categorization allowed us to quantify the additive interaction effect between metformin use and OS levels using the indicator of Relative Excess Risk due to Interaction (RERI), as recommended by the STROBE statement [18]. The RERI was computed using the formula RERI = RR_11_ − RR_10_ − RR_01_ + 1, where RR_11_ represents the relative risk for those exposed to both factors (metformin nonuser + high OS), RR_10_ for exposure to one factor (metformin user + high OS), and RR01 for exposure to the other factor. The 95% CI for these estimations was derived using the delta method described by Hosmer and Lemeshow [19]. This analysis was adjusted for the same covariates as in the fully adjusted multivariable model above.

A series of sensitivity analyses were conducted to assess the robustness of the findings. First, we excluded deaths occurring in the first year of follow-up to minimize the potential for reverse causation. Second, accidental death was applied as a negative control outcome (deaths from “Accidents (unintentional injuries) (V01-X59, Y85-Y86)”). Third, considering that patients taking metformin all had comorbid diabetes, we examined the impact of sulfonylureas, another commonly used medication for cancer survivors with type 2 diabetes mellitus (T2DM) in our study cohort, on cardiometabolic outcomes to rule out the confounding effect of the presence of T2DM. Fourth, we further adjusted for the following covariates: variates reflecting the severity of diabetes (HbA1c, diabetic retinopathy) and the use of glucose-lowering medications with potential cardiometabolic benefits including GLP-1 receptor agonists and SGLT-2 inhibitors. Fifth, patients who underwent renal dialysis within the past 12 months were excluded from the analysis. Lastly, our study was conducted across the overall population and within specific subgroups stratified by age, gender, BMI, and race.

## Results

In this study involving 3995 cancer survivors (representative of a national weighted population of 21,671,061 individuals, weighted mean [SE] age, 62.62 [0.33] years, 53.04% women), the ethnic composition was diverse. The majority, 2727 participants (68.26%), identified as Non-Hispanic White. Five hundred eighty five individuals (14.64%) were Non-Hispanic Black, 260 (6.51%) were Mexican American, 220 (5.51%) were of other Hispanic backgrounds, and 203 (5.08%) belonged to other racial groups, including American Indian/Native Alaskan/Pacific Islander, Asian, and multiracial categories. There were a total of 3547 metformin nonusers and 448 metformin users. Table 1 presents the baseline profile of these participants, categorized based on their usage of metformin.

**Table 1:** Sample size and characteristics of metformin user and nonuser groups among US cancer survivors, NHANES 2003 to 2018

Variable	Study population		
	All	Metformin nonusers	Metformin users
Age group, y			
<45	393 (9.84)	384 (13.25)	9 (3.08)
45 to 59	715 (17.9)	660 (26.21)	55 (19.23)
≥60	2887 (72.27)	2503 (60.54)	384 (77.69)
Gender			
Women	2119 (53.04)	1893 (58.22)	226 (46.64)
Men	1876 (46.96)	1654 (41.78)	222 (53.36)
Race and ethnicity			
Non-Hispanic White	2727 (68.26)	2475 (86.42)	252 (81.63)
Non-Hispanic Black	585 (14.64)	496 (5.29)	89 (8.06)
Mexican American	260 (6.51)	210 (2.24)	50 (4.16)
Other Race	203 (5.08)	176 (3.78)	27 (3.87)
Other Hispanic	220 (5.51)	190 (2.27)	30 (2.27)
Educational level			
<High school	395 (9.89)	329 (4.59)	66 (7.63)
High school	1418 (35.49)	1260 (30.84)	158 (30.04)
>High school	2182 (54.62)	1958 (64.56)	224 (62.33)
Family poverty income ratio			
<1.30	1000 (25.03)	871 (15.59)	129 (17.89)
1.30 to 3.49	1628 (40.75)	1426 (35.57)	202 (42.79)
≥3.5	1367 (34.22)	1250 (48.84)	117 (39.32)
Weight status, BMI			
<25	1128 (28.24)	1067 (31.08)	61 (9.77)
25 to 30	1406 (35.19)	1262 (35.33)	144 (26.79)
≥30	1461 (36.57)	1218 (33.59)	243 63.45)
Smoking			
Never	1804 (45.16)	1595 (45.81)	209 (44.72)
Former	1570 (39.3)	1377 (37.35)	193 (44.50)
Now	621 (15.54)	575 (16.85)	46 (10.78)
Alcohol use			
Never	558 (13.97)	484 (10.94)	74 (12.07)
Former	991 (24.81)	852 (19.27)	139 (25.38)
Mild	1642 (41.1)	1462 (44.24)	180 (48.08)
Moderate	439 (10.99)	406 (14.57)	33 (7.17)
Heavy	365 (9.14)	343 (10.97)	22 (7.30)
Physical activity, MET-h/wk			
Low intensity PA	2879 (72.07)	2529 (69.06)	350 (80.15)
High intensity PA	1116 (27.93)	1018 (30.94)	98 (19.85)
Hyperlipidemia			
No	873 (21.85)	823 (22.50)	50 (8.87)
Yes	3122 (78.15)	2724 (77.50)	398 (91.13)
Diabetes			
No	3135 (78.47)	3135 (91.21)	0 (0.00)
Yes	860 (21.53)	412 (8.79)	448 (100.00)
Depression, PHQ9			
[0,9]	3578 (89.56)	3193 (91.13)	385 (88.73)
[10,27]	417 (10.44)	354 (8.87)	63 (11.27)
Antihyperlipidemic drug use			
No	2483 (62.15)	2320 (68.16)	163 (33.30)
Yes	1512 (37.85)	1227 (31.84)	285 (66.70)
Antihypertensive drug use			
No	1701 (42.58)	1633 (52.76)	68 (15.26)
Yes	2294 (57.42)	1914 (47.24)	380 (84.74)
Years since 1st cancer diagnosis, y	11.91 (0.26)	11.91 (0.27)	11.94 (0.70)

*BMI* Body Mass Index (calculated as weight in kilograms per meter squared), *y* years, *h/wk* hours per week, *PHQ9* Patient Health Questionnaire-9, *NHANES* the National Health and Nutrition Examination Survey

^a^Weighted to be nationally representative. The weighted percentage may not sum to 100% due to missing data

Among 3995 cancer survivors, a total of 1233 deaths occurred during a median follow-up of 6.42 years (77 months), including 481 deaths from cardiometabolic diseases. Table 2 illustrates the notable reduction in both all-cause and cardiometabolic mortality among cancer survivors undergoing metformin therapy. In the minimally adjusted model, hazard ratios (HRs) with 95% CIs for all-cause and cardiometabolic mortality were 0.60 (95% CI, 0.45–0.81) and 0.62 (95% CI, 0.42–0.93), respectively. Upon further adjustments in the fully adjusted model for covariates, the HRs for all-cause and cardiometabolic mortality were 0.62 (95% CI, 0.47–0.81, E-value = 2.61) and 0.65 (95% CI, 0.44–0.97, E-value = 2.45), respectively, among cancer survivors receiving metformin treatment compared to nonusers.

**Table 2:** Association of metformin use with all-cause and cardiometabolic mortality risk among US cancer survivors, NHANES 2003 to 2018

Mortality outcome	Death/No.	Hazard ratio (95% CI)			
		Minimally adjusted model^a^	*P* value	Fully adjusted model^b^	*P* value
**All-cause mortality**					
Treatment group					
Metformin nonuser	1104/3547	1 [Reference]		1 [Reference]	
Metformin user^c^	129/448	0.60 (0.45, 0.81)	<0.001	0.62 (0.47, 0.81)	<0.001
**Cardiometabolic mortality**					
Treatment group					
Metformin nonuser	410/3547	1 [Reference]		1 [Reference]	
Metformin user	71/448	0.62 ( 0.42, 0.93)	0.021	0.65 (0.44, 0.97)	0.033

*Abbreviations*: *BMI* body mass index (defined as weight in kilograms divided by height in meters squared), *CI* Confidence interval, *NHANES* the National Health and Nutrition Examination Survey

^a^Minimally adjusted model: Adjusted for age, gender, race/ethnicity, educational level

^b^Fully adjusted model: Further adjusted for family poverty income ratio, BMI, smoking status, alcohol use, physical activity, hyperlipidemia, diabetes, depression, antihyperlipidemic drug use, antihypertensive drug use, and years since the first cancer diagnosis

^c^Metformin users were identified based on self-reported prescription medication use of metformin in the past month during household interviews. Nonusers were those who did not report using metformin

In our study cohort, 981 participants experienced cardiovascular diseases (CVDs), representing a weighted prevalence of 19.66% (95% CI, 17.64–21.69%). Furthermore, stroke occurred in 356 participants (weighted prevalence: 6.54% [95% CI, 5.51–7.56%]), hypertension in 2885 (weighted prevalence: 67.96% [95% CI, 63.49–72.43%]), and coronary heart disease in 390 (weighted prevalence: 8.01% [95% CI, 6.81–9.22%]). Among these cancer survivors, a total of 448 individuals were identified as metformin users. Survey-weighted logistic regression analysis on the cross-sectional data in Table 3 showed an inverse association between metformin use and cardiometabolic diseases in cancer survivors. The odds ratio (OR) for the incidence of total CVD in those undergoing metformin use, compared to those not receiving it, was 0.41 (95% CI, 0.28–0.59) after full adjustment. The ORs for the outcomes of stroke, hypertension, and coronary heart disease were 0.44 (95% CI, 0.26–0.74), 0.27 (95% CI, 0.14–0.52), and 0.41 (95% CI, 0.21–0.78), respectively, in the fully adjusted model. The E-values ranged from 2.49 to 4.31, which indicated that an unmeasured confounder would need to have at least an OR of 2.49 to explain the observed associations.

**Table 3:** Correlations of metformin use with four specific cardiometabolic diseases risk among US cancer survivors, NHANES 2003 to 2018

Cardiometabolic comorbidities	Event/No.	Odds ratio (95% CI)			
		Minimally adjusted model^a^	*P* value	Fully adjusted model^b^	*P* value
**Total CVD**					
Treatment group					
Metformin nonuser	839/3547	1 [Reference]		1 [Reference]	
Metformin user	142/448	0.47 (0.33,0.67)	<0.001	0.41 (0.28,0.59)	<0.001
**Stroke**					
Treatment group					
Metformin nonuser	308/3547	1 [Reference]		1 [Reference]	
Metformin user	48/448	0.46 (0.27,0.77)	0.004	0.44 (0.26,0.74)	0.002
**Hypertension**					
Treatment group					
Metformin nonuser	2518/3547	1 [Reference]		1 [Reference]	
Metformin user	367/448	0.48 (0.27,0.85)	0.012	0.27 (0.14, 0.52)	<0.001
**Coronary heart disease**					
Treatment group					
Metformin nonuser	335/3547	1 [Reference]		1 [Reference]	
Metformin user	55/448	0.49 (0.26, 0.90)	0.021	0.41 (0.21,0.78)	0.007
*Abbreviations*: *BMI* body mass index (defined as weight in kilograms divided by height in meters squared), *CI* Confidence interval, *CVD* Cardiovascular disease, *NHANES* the National Health and Nutrition Examination Survey					

^a^Minimally adjusted model: Adjusted for age, gender, race/ethnicity, educational level

^b^Fully adjusted model: Further adjusted for family poverty income ratio, BMI, smoking status, alcohol use, physical activity, hyperlipidemia, diabetes, depression, antihyperlipidemic drug use, antihypertensive drug use, and years since the first cancer diagnosis

**Fig. 2:**
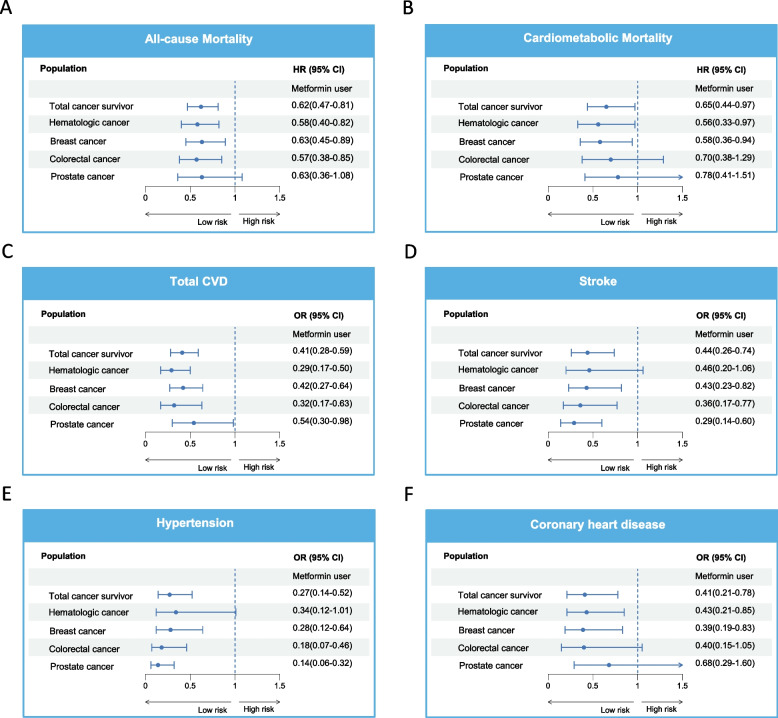
Association of metformin use with the risk of all-cause mortality (**A**), cardiometabolic mortality (**B**), and cardiometabolic diseases (**C**-**F**) in the overall cohort of cancer survivors and four specific cancer subgroups with high cardiometabolic risk. Metformin nonusers group was defined as the reference. Hazard ratios (depicted by solid symbols) with corresponding 95% CIs (represented by error bars) of metformin use for all-cause mortality (**A**) and cardiometabolic mortality (**B**) were estimated using weighted multivariable Cox regression models. Odds ratios (indicated by solid symbols) with corresponding 95% CIs (represented by error bars) of metformin use for the total cardiovascular diseases (**C**), stroke (**D**), hypertension (**E**), and coronary heart disease (**F**) were estimated using weighted multivariable logistic regression models. Both the multivariable Cox and logistic regression models were adjusted for age, gender, race/ethnicity, educational level, family poverty income ratio, BMI, smoking status, alcohol use, physical activity, hyperlipidemia, diabetes, depression, antihyperlipidemic drug use, antihypertensive drug use, and years since the first cancer diagnosis. HR, Hazard Ratio; OR, Odds Ratio; CI, Confidence interval; CVD, Cardiovascular disease; BMI, body mass index

Previous research has indicated an elevated risk of cardiometabolic diseases in patients with certain types of tumors. In our study involving a nationally representative cohort of cancer survivors, the inverse association between cardiometabolic risk and metformin use was validated in specific cancer types. Fully adjusted analyses, as illustrated in Fig. 2, show a reduction in all-cause mortality risk in survivors of hematologic (HR, 0.58; 95% CI, 0.40–0.82), breast (HR, 0.63; 95% CI, 0.45–0.89), and colorectal (HR, 0.57; 95% CI, 0.38–0.85) cancers treated with metformin. Decreased risk of cardiometabolic mortality was also noted in survivors of hematologic (HR, 0.56; 95% CI, 0.33–0.97) and breast (HR, 0.58; 95% CI, 0.36–0.94) cancers, but not among those with colorectal (HR, 0.70; 95% CI, 0.38–1.29) and prostate (HR, 0.78; 95% CI, 0.41–1.51) cancers when compared to individuals without metformin use. Regarding the risk of cardiometabolic diseases, a significant reduction in total CVD risk was observed in patients with all four specific cancer types treated with metformin. In breast, colorectal, and prostate cancer survivor subgroups, metformin demonstrated significantly beneficial effects on the risks of stroke and hypertension, with the protective effect of metformin against coronary heart disease primarily observed in survivors of hematologic and breast cancers.

Oxidative stress is a known hallmark of cardio-oncology, and metformin plays an important role in regulating the oxidant-antioxidant system. We further investigated whether antioxidant properties could explain the inverse relationship between metformin use and cardiometabolic risk in cancer survivors. Participants’ exposure to OS-related damage was evaluated by oxidative balance scores, and the interaction analysis was conducted to examine the antagonistic effect of metformin on OS. The interaction effects between metformin use and oxidative stress levels on all the cardiometabolic outcomes are shown in the Table 4. Compared to cancer survivors with metformin use and low OS level (reference), low-OS survivors without metformin use (“single-hit”), and high-OS survivors with metformin use (“single-hit”), those with no metformin usage but in high OS level (“double-hit”) exhibited the highest risks of all-cause/cardiometabolic mortality and cardiometabolic diseases (Table 4). A significant additive interaction was observed in the outcomes of all-cause mortality (RERI, 0.47; 95% CI, 0.21 to 0.73), cardiometabolic mortality (RERI, 0.53; 95% CI, 0.24 to 0.82), total CVD (RERI, 0.29; 95% CI, 0.06 to 0.52), stroke (RERI, 0.79; 95% CI, 0.28 to 1.30), and hypertension (RERI, 0.66; 95% CI, 0.09 to 1.23).

**Table 4:** Relative excess risk of all-cause mortality, cardiometabolic mortality, and specific cardiometabolic diseases due to antagonistic interaction effect of metformin use and oxidative stress levels in cancer survivors

	**All-cause mortality**		**Cardiometabolic mortality**		**Total CVD**	
	**Event/No.**	**HR** **(95%CI)**^a^	**Event/No.**	**HR** **(95%CI)**^a^	**Event/No.**	**OR** **(95%CI)**^a^
Metformin user with low OS	57/201	1.00 (ref)	32/201	1.00 (ref)	51/201	1.00 (ref)
Metformin nonuser with low OS	478/1747	1.36 (0.93, 1.98)	177/1747	1.27 (0.75, 2.17)	338/1747	2.59 (1.58,4.26)
Metformin user with high OS	72/247	0.95 (0.59, 1.55)	39/247	1.03 (0.58, 1.84)	91/247	1.50 (0.85,2.65)
Metformin nonuser with high OS	626/1800	1.78 (1.23, 2.57)	233/1800	1.83 (1.14, 2.93)	501/1800	3.38 (2.07,5.52)
**RERI**		**0.47** **(0.21, 0.73)**		**0.53** **(0.24, 0.82)**		**0.29** **(0.06,0.52)**
	**Stroke**		**Hypertension**		**Coronary heart disease**	
	**Event/No.**	**OR** **(95%CI)**^a^	**Event/No.**	**OR** **(95%CI)**^a^	**Event/No.**	**OR** **(95%CI)**^a^
Metformin user with low OS	18/201	1.00 (ref)	161/201	1.00 (ref)	23/201	1.00 (ref)
Metformin nonuser with low OS	102/1747	1.94 (0.96,3.93)	1182/1747	3.15 (1.46, 6.78)	149/1747	2.75 (1.28,5.91)
Metformin user with high OS	30/247	1.22 (0.52,2.84)	206/247	0.81 (0.31, 2.11)	32/247	1.58 (0.71,3.49)
Metformin nonuser with high OS	206/1800	2.95 (1.55,5.60)	1336/1800	3.62 (1.69, 7.75)	186/1800	3.44 (1.56,7.58)
**RERI**		**0.79** **(0.28, 1.30)**		**0.66** **(0.09, 1.23)**		**0.11** **(-0.13, 0.35)**

*Abbreviations*: *HR* Hazard Ratio, *OR* Odds Ratio, *CI* Confidence interval, *RERI* Relative excess risk due to interaction, *OS* Oxidative stress, *CVD* Cardiovascular disease, *CHD* Coronary heart disease, *BMI* body mass index

^a^Both multivariable Cox and logistic regression models were adjusted for age, gender, race/ethnicity, educational level, family poverty income ratio, BMI, smoking status, alcohol use, physical activity, hyperlipidemia, diabetes, depression, antihyperlipidemic drug use, antihypertensive drug use, and years since the first cancer diagnosis

In the stratified analyses detailed in Supplementary Table 2 (Additional file 1: Table S2), based on gender, race, and baseline BMI, the cardiometabolic protective influence of metformin on the risk of total CVD, hypertension, and CHD was more significant among older individuals. The study revealed no significant variations in the negative relationship of metformin use with cardiometabolic outcomes across diverse genders, races, or obesity categories. The robustness of our findings was further validated by performing a series of sensitivity analyses. In order to minimize the potential reverse causation, we excluded deaths that occurred within the initial 1-year follow-up period and the results remained significant (Additional file 1: Table S3, 4). The analysis of the negative control outcome of accidental death revealed the absence of significant association of metformin use and the risk of accidental death (*N* = 34; HR, 2.59; 95% CI, 0.27–24.78). When sulfonylureas use, instead of metformin use, was considered as the exposure, the correlations with all-cause mortality and cardiometabolic outcomes were non-significant (Additional file 1: Table S5, 6). Further adjustments for HbA1c, diabetic retinopathy, GLP-1 receptor agonist use, and SGLT-2 inhibitor use did not substantially alter the results (Additional file 1: Table S7, 8). After excluding participants who underwent renal dialysis within the past 12 months, the robust inverse correlation between cardiometabolic risk and metformin use remained evident (Additional file 1: Table S9, 10).

## Discussion

In this study, conducted within a nationally representative cohort of cancer survivors in the USA, with a median follow-up duration of 6.4 years, metformin use as compared to non-use was inversely associated with the risk of cardiometabolic diseases, all-cause mortality, and cardiometabolic mortality in cancer survivors. This inverse association was observed not only in the overall population of cancer survivors, but also in patients with specific cancer types associated with a higher cardiometabolic risk. The potential mechanisms underlying this inverse association of metformin were further explored. Considering the pivotal role of oxidative stress in cardio-oncology and the widely reported antioxidative capacity of metformin, the OBS system was implemented for a quantitative assessment of oxidative stress levels within our cohort. The findings suggested *t*hat metformin use might exert cardiometabolic protection in patients with cancer by antagonizing oxidative stress. These findings remained consistent across diverse clinical subgroups and were corroborated by sensitivity analyses.

### Challenges in managing cardiometabolic risks among cancer survivors

In the current landscape of oncological advancements, the USA has witnessed an increase in the number of cancer survivors, reaching nearly 17 million, many of whom continued to receive long-term cancer treatment post-diagnosis. Despite extended survival and reduced mortality due to cancer treatment innovations, the growing burden of CMD and associated mortality risks among cancer survivors is gaining attention. Early screening and treatment approaches in breast cancer cohort have elevated the 5-year survival rate above 90% [20]. However, this is contrasted sharply by an increased risk of heart disease, diabetes, and cardiovascular-related deaths in these patients [21, 22]. A common reason is the reduced adherence to cardiometabolic medications following cancer diagnosis. Research indicated a notable decline in statin adherence in patients with breast cancer, from 67% pre-diagnosis to just 35% 2 years post-diagnosis, which was also evident in antihypertensive and antidiabetic medications [23, 24]. Furthermore, treatment regimens for breast cancer, such as anthracycline therapy and endocrine therapy, have been associated with heightened adverse effects on cardiac well-being and metabolic diseases [22, 25]. These factors, coupled with reduced physical activity during cancer treatment contributed to a detrimental cycle that heightens CMD risk in patients with breast cancer [26, 27]. This cycle is recognized as prevalent across various genders and cancer types. Addressing and actively preventing the potential CMD risk in patients with cancer is crucial, not only for cancer cure or chronic management but also for maximizing long-term health and productivity.

### Interplay of cancer and cardiometabolic diseases: the key role of oxidative stress

Cancer and cardiometabolic diseases are intricately linked and mutually exacerbating. Specifically, the treatment pattern and lifestyle factors in patients with cancer can elevate the risk of CMD, as discussed above. And vice versa, an elevated risk of cancer incidence and cancer-related mortality has also been observed in populations with cardiometabolic diseases [28]. These two conditions appear to share common mechanisms related to metabolic disorders [29]. The “Warburg effect” underscores that cancer cells display a distinct metabolic phenotype, marked by augmented glucose uptake compared to normal cells. Advances in sequencing technology have also unveiled the significant role of metabolic dysregulation in tumor growth and metastasis. The observed metabolic abnormalities, such as fumarate in renal cell carcinoma, glycine in breast cancer suggested that somatic mutations may emerge as downstream effects of disruptions in cellular energy metabolism [30, 31].

Existing research has highlighted the pivotal role of oxidative stress in the field of Cardio-oncology. The cancer metabolic theory posited that the crux of metabolic dysregulation in cancer centers on defects in mitochondrial oxidative phosphorylation (OXPHOS) [32]. Impaired OXPHOS complements glycolysis in tumor metabolism. Concurrently, decreased respiratory efficiency in the OXPHOS pathway produces heightened reactive oxygen species (ROS). This resulting oxidative stress, characterized by elevated ROS levels, possesses notable mutagenic and carcinogenic properties. Oxidative stress increases the mutation rate in cells with for its DNA-damaging capacity [33]. Furthermore, chemotherapeutic agents like doxorubicin have also been reported to exhibit substantial cardiotoxicity via oxidative stress [34]. Shifting the focus to cardiometabolic diseases, an imbalance between oxidants and antioxidants is also a common characteristic. Markers of redox imbalance were found to be elevated in models of hypertension [35, 36]. Moreover, Niemann et al. observed increased OS markers in cardiomyocytes of patients undergoing coronary artery bypass graft surgery [37].

In summary, oxidative stress emerges as a significant potential comorbid mechanism in the relationship between cancer and cardiometabolic diseases. Interventions targeting oxidative stress might play a key role in breaking the vicious cycle of “cancer-cardiometabolic disease” interplay.

### Metformin: a potential game-changer in cardiometabolic disease management among cancer survivors

Metformin, acknowledged as a crucial first-line agent in managing T2DM, primarily functions by activating AMPK pathway in cells and curtailing hepatic glucose production. Beyond its conventional hypoglycemic efficacy, the pharmacological versatility of metformin across various human systems has garnered significant clinical interest. Apart from regulating glucose and lipid metabolism in cardiomyocytes, metformin lowered advanced glycation end products and ROS levels in the endothelium, offering substantial protection for cardiometabolic health [38]. Ongoing clinical trials affirmed metformin’s beneficial impact on diverse cardiometabolic outcomes in diabetic population, including coronary death, primary cardiovascular disease, body weight, and so on [39]. Furthermore, Zheng et al., by assessing the genetically proxied effects of metformin targets on a comprehensive array of cardiometabolic outcomes, have demonstrated its effectiveness in improving cardiometabolic conditions such as CHD, BMI, and blood pressure in the general population [40].

Apart from its prominent regulatory role in CMD, metformin may be promising in combating malignancies in organs such as the breast, kidney, and endometrium. Although its anti-carcinogenic effect has yet to be proven clinically [41], properties found in preclinical studies, including inhibiting growth and inducing cell death of cancerous cells, supported its anticancer potential [42]. With the increasing risk of CMD in the cancer survivors, it is regrettable that medication targeting the underlying mechanisms has yet gained approval for treatment. Our research findings suggested that metformin might be crucial in disrupting the detrimental “cancer-cardiometabolic disease” cycle. Findings from our research revealed that patients with cancer using metformin experience significant reductions in the risk of all-cause mortality and those associated with CMD and related mortality, when compared to those not using metformin.

To further evaluate the robustness of all regression models, E-value was also applied to explore the impact of unmeasured confounding variables on our findings. The E-values of all-cause and cardiometabolic mortality outcomes were 2.61 and 2.45, respectively. These findings implied that an unobserved confounder need to exhibit stronger associations with both metformin use and all-cause/cardiometabolic mortality than the measured confounder (HR of the confounder diabetes were 1.56 and 2.38, respectively), in order to fully explain away the observed HR of metformin use. The same was true for E-values of specific cardiometabolic diseases. The existence of an unobserved confounder that would have a stronger association with both metformin use and cardiometabolic outcomes than the confounder diabetes seems unlikely.

### Oxidative stress mechanism and robust cardiometabolic protection across cancer subgroups

The mechanisms through which metformin intervenes to modulate cardiometabolic risk among cancer survivors were further investigated. As mentioned earlier, oxidative stress is a key shared pathogenic mechanism in both cancer and cardiometabolic diseases. Previous studies have already confirmed the potential of metformin in counteracting oxidative stress [43–45]. Expanding on our research affirming the protective impact of metformin on cardiometabolic health of cancer survivors, the OBS was utilized to assess OS levels in these individuals quantitatively. Subsequent interaction analysis revealed that metformin enhances cardiometabolic outcomes by exerting an antagonistic effect on the pathological process of oxidative stress. This observation shed light on the potential mechanism underlying metformin’s cardiometabolic protective properties.

In our comprehensive cancer-patient cohort, metformin was found to significantly reduce the risks of all-cause mortality, cardiometabolic mortality, and the specific risk of cardiometabolic diseases. However, the risk of subsequent CMD varied among patients with different cancer types. A recent cohort study involving 126,120 cancer survivors indicated an increased risk of cardiovascular events, such as CHD and stroke. Subgroup analysis further revealed elevated cardiovascular event risks in patients with hematologic malignancies and increased stroke risks in patients with breast cancer [46]. Another study of cancer survivors in UK Biobank suggested the highest hypertension comorbidity risk in prostate and colorectal cancer subgroups [47]. A subgroup analysis was conducted on patients with hematologic cancer, breast cancer, colorectal cancer, and prostate cancer who are at high risk for the CMD as mentioned above. The results of subgroup analysis similarly supported that metformin effectively reduces the risk of CMD and associated mortality risks in these specific cancer subgroups.

### Strengths and limitation

As our understanding of the pathogenesis and treatment principles of cancer deepens, the therapeutic objectives for cancer survivors are evolving beyond merely “curing” cancer, aiming instead to foster a prolonged and productive lifestyle. With increasing oncologic survival, cancer survivors are at growing risk for various chronic conditions. Effective management of cancer survivors with concurrent CMD is critically essential. However, no medication has been widely recognized as effective in treating CMD among cancer survivors. Our current study addressed gaps in existing literature concerning the pharmacological management of cardiometabolic comorbidities in cancer survivors. It offered concrete evidence linking metformin use to enhanced cardiometabolic health post-cancer. One key strength of our study lies in the novel finding that metformin significantly reduces the risk of cardiometabolic diseases, all-cause mortality, and cardiometabolic mortality in cancer survivors. Moreover, even within subgroups of patients with four cancer types at higher cardiometabolic risk, the robust protective effect of metformin on cardiometabolic outcomes persisted. Crucially, our analysis utilized a large, nationally representative sample of cancer survivors in the USA, encompassing cancers with relatively high 5-year survival rates, such as breast, colorectal, and prostate cancers, and those with less favorable outcomes, including hematologic and ovarian cancers. This diverse representation enhances the clinical applicability of our findings, offering promising aspects for CMD treatment in cancer survivors with metabolic vulnerability and suggesting a new avenue for expanding the therapeutic spectrum of the classic drug metformin. Lastly, by systematically assessing oxidative stress levels in patients and conducting interaction analyses with metformin use, our results elucidated the potential pharmacological mechanism of metformin’s cardiometabolic protective effect by antagonizing oxidative stress in cancer survivors. This insight holds significance for future interventional target of oxidative stress and the development of CMD treatments among patients with cancer.

Despite these strengths, our study has several limitations that merit consideration. First, due to the observational nature of the NHANES database, it was challenging to incorporate all residual covariates into the adjusted model. However, we determined E-values to illustrate that the influence of unmeasured confounding factors is unlikely to be sufficient to nullify the observed correlations. Second, although adjustments for tumor-related factors, such as “years since the first cancer diagnosis,” were incorporated, NHANES lacked detailed data on specific cancer staging and treatment.

Third, information on cardiometabolic disease outcomes in this study was derived from cross-sectional data. Therefore, we could not definitively ascertain the temporal relationship between metformin use and reported cardiometabolic diseases. We also concur that reliance on self-reported data for cardiometabolic disease outcomes and cancer status might lead to misclassification, which possibly resulted in non-differential or differential bias. Non-differential misclassification, which occurs when the misclassification of cardiometabolic outcome is independent of the exposure status, could potentially attenuate the observed correlations towards the null. In certain cases, this bias may lead to false positive results, especially when the misclassification interacts with other variables. Although the NHANES survey employed a stringent data collection protocol to minimize misclassification, we cannot fully rule out the possibility of misclassification. To mitigate this concern, future studies could incorporate medical records or clinical assessments to validate the self-reported diagnoses, thereby reducing the likelihood of misclassification and providing more precise correlation estimation.

Fourth, another limitation of this study was that we only had binary information on whether participants used metformin or not, but lacked data on the duration of medication use, dosage, and other relevant details. Fifth, information on metformin use (exposure) was obtained by reviewing participants’ medication use in the past month during the household interview. However, the NHANES database lacked survival data of cardiometabolic outcomes for the period between metformin treatment initiation and the start of the follow-up (household interview). Consequently, our findings were possibly influenced by immortal time bias. Although we conducted a sensitivity analysis by excluding cancer survivors who died early in the follow-up period and found that the results remained robust, the potential impact of immortal time bias should be considered when applying our findings.

Sixth, due to the strong association of the exposure with the negative control outcome (i.e., accidental deaths), we cannot exclude the possibility of a certain degree of bias in our main analyses. Lastly, the oxidative balance score was computed based on the assumption that all prooxidants and antioxidants linearly correlated with oxidative stress levels, without considering the threshold effect of antioxidants. Studies have indicated that specific antioxidants, such as carotenoids and copper, might demonstrate prooxidative effects when administered in elevated concentrations. Future research should consider these factors or introduce more robust biomarkers related to oxidative stress to provide more reliable validation of the cardiometabolic protective mechanisms of metformin.

## Conclusions

In this prospective cohort study encompassing a nationally representative sample of US cancer survivors, metformin use as compared to non-use was inversely associated with the risks of all-cause mortality, cardiometabolic disease, and associated mortality. This study provided novel evidences and perspectives on the pharmaceutical management of cancer survivors in the field of cardio-oncology, highlighting potential directions for the design and development of cardiometabolic protective drugs specifically beneficial for cancer populations. Considering the lack of detailed data on specific cancer staging and treatment as well as the cross-sectional nature of cardiometabolic disease outcomes, subsequent longitudinal and more comprehensive studies are urgently needed to further elucidate the practical application and pharmacological mechanisms of metformin in the management of cardiometabolic health among cancer survivors.

### Supplementary Information

Additional file 1: Figure S1. The hypothetical directed acyclic graph used to select potential covariates. Table S1. Components of the oxidative balance score. Table S2. Association of Metformin Use with All-Cause Mortality and Cardiometabolic Outcomes Among US Cancer Survivors by Age, Gender, BMI, and Race, NHANES 2003 to 2018. Table S3. Association of Metformin Use with All-Cause and Cardiometabolic Mortality Risk Among US Cancer Survivors, NHANES 2003 to 2018. Table S4. Relative excess risk of all-cause mortality, cardiometabolic mortality due to antagonistic interaction effect of metformin use and oxidative stress levels in cancer survivors. Table S5. Association of Sulfonylurea Use with All-Cause and Cardiometabolic Mortality Risk Among US Cancer Survivors, NHANES 2003 to 2018. Table S6. Correlations of Sulfonylurea Use with Four Specific Cardiometabolic Diseases Risk Among US Cancer Survivors, NHANES 2003 to 2018. Table S7. Association between Metformin Use and All-Cause/Cardiometabolic Mortality Risk with further adjustment of HbA1c, Diabetic Retinopathy, GLP-1 Receptor Agonists Use and SGLT-2 Inhibitors Use. Table S8. Correlations between Metformin Use and Four Specific Cardiometabolic Diseases Risk with further adjustment of HbA1c, Diabetic Retinopathy, GLP-1 Receptor Agonists Use and SGLT-2 Inhibitors Use. Table S9. Association between Metformin Use and All-Cause/Cardiometabolic Mortality Risk Among US Cancer Survivors after Excluding Patients Receiving Dialysis in Past 12 Months, NHANES 2003 to 2018. Table S10. Correlations between Metformin Use and Four Specific Cardiometabolic Diseases Risk Among US Cancer Survivors after Excluding Patients Receiving Dialysis in Past 12 Months, NHANES 2003 to 2018.

## Data Availability

The NHANES data supporting the results of this study are available online through https://wwwn.cdc.gov/nchs/nhanes/Default.aspx.

## Abbreviations

95% CI: 95% Confidence intervals
AMPK: Adenosine 5’ monophosphate-activated protein kinase
BMI: Body mass index
CHD: Coronary heart disease
CMD: Cardiometabolic disease
CS: Cancer survivors
CVDs: Cardiovascular diseases
DAG: Directed acyclic graph
HR: Hazard ratio
ICD-10: The tenth revision of the international classification of diseases
MET: Metabolic equivalent
NHANES: National health and nutrition examination survey
OBS: Oxidative balance score
OR: Odds ratio
OS: Oxidative stress
OXPHOS: Oxidative phosphorylation
PAQ: Physical activity questionnaire
PHQ9: Patient health questionnaire-9
PIR: Poverty to income ratio
RERI: Relative excess risk due to interaction
ROS: Reactive oxygen species
SE: Standard error of mean
T2DM: Type 2 diabetes mellitus
