# Supplementary material for: Metformin use correlated with lower risk of cardiometabolic diseases and related mortality among US cancer survivors: evidence from a nationally representative cohort study
Source: BMC Med. 2024 Jun 26;22:269. doi: 10.1186/s12916-024-03484-y (PMC11210152; doi:10.1186/s12916-024-03484-y)
Supplement: Supplementary file 1 — Additional file 1: Figure S1. The hypothetical directed acyclic graph used to select potential covariates. Table S1. Components of the oxidative balance score. Table S2. Association of Metformin Use with All-Cause Mortality and Cardiometabolic Outcomes Among US Cancer Survivors by Age, Gender, BMI, and Race, NHANES 2003 to 2018. Table S3. Association of Metformin Use with All-Cause and Cardiometabolic Mortality Risk Among US Cancer Survivors, NHANES 2003 to 2018. Table S4. Relative excess risk of all-cause mortality, cardiometabolic mortality due to antagonistic interaction effect of metformin use and oxidative stress levels in cancer survivors. Table S5. Association of Sulfonylurea Use with All-Cause and Cardiometabolic Mortality Risk Among US Cancer Survivors, NHANES 2003 to 2018. Table S6. Correlations of Sulfonylurea Use with Four Specific Cardiometabolic Diseases Risk Among US Cancer Survivors, NHANES 2003 to 2018. Table S7. Association between Metformin Use and All-Cause/Cardiometabolic Mortality Risk with further adjustment of HbA1c, Diabetic Retinopathy, GLP-1 Receptor Agonists Use and SGLT-2 Inhibitors Use. Table S8. Correlations between Metformin Use and Four Specific Cardiometabolic Diseases Risk with further adjustment of HbA1c, Diabetic Retinopathy, GLP-1 Receptor Agonists Use and SGLT-2 Inhibitors Use. Table S9. Association between Metformin Use and All-Cause/Cardiometabolic Mortality Risk Among US Cancer Survivors after Excluding Patients Receiving Dialysis in Past 12 Months, NHANES 2003 to 2018. Table S10. Correlations between Metformin Use and Four Specific Cardiometabolic Diseases Risk Among US Cancer Survivors after Excluding Patients Receiving Dialysis in Past 12 Months, NHANES 2003 to 2018. [file 12916_2024_3484_MOESM1_ESM.doc]

Figure S1. The hypothetical directed acyclic graph used to select potential covariates ^a^


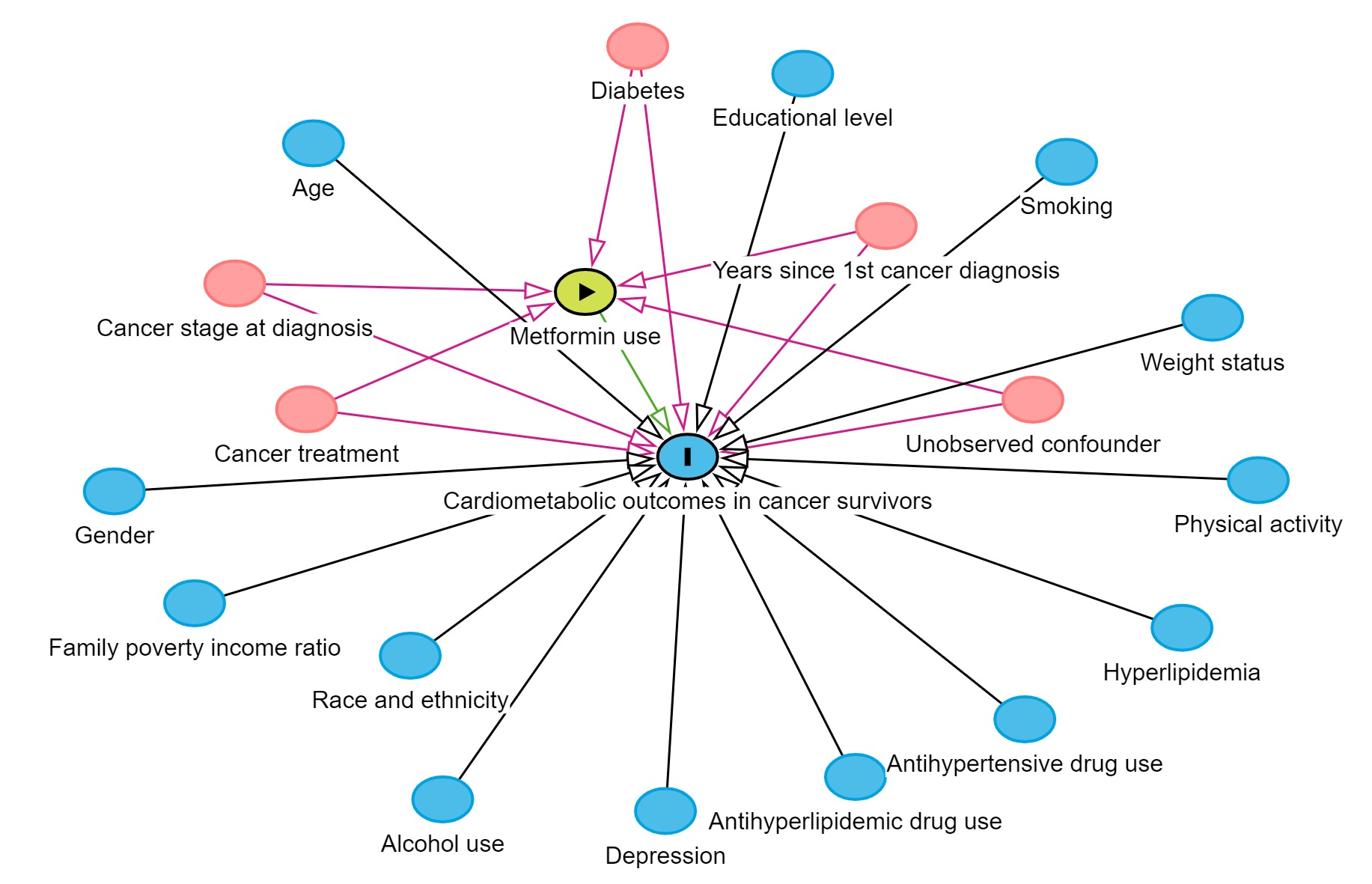


^a^ To facilitate reading, the arrows from blue circles to Metformin use are not shown.

Table S1. Components of the oxidative balance score

| **OBS components** | Property | Male | | | Female | | |
| --- | --- | --- | --- | --- | --- | --- | --- |
|  |  | 0 | 1 | 2 | 0 | 1 | 2 |
| **Dietary OBS components** |  |  |  |  |  |  |  |
| Dietary fiber (g/d) | A | <12.56 | 12.56-19.70 | ≥19.70 | <10.10 | 10.10-16.31 | ≥16.31 |
| Carotene (RE/d) | A | <98.83 | 98.83-306.25 | ≥306.25 | <98.08 | 98.08-383.50 | ≥383.50 |
| Riboflavin (mg/d) | A | <1.79 | 1.79-2.69 | ≥2.69 | <1.34 | 1.34-2.02 | ≥2.02 |
| Niacin (mg/d) | A | <20.65 | 20.65-29.75 | ≥29.75 | <14.52 | 14.52-21.86 | ≥21.86 |
| Vitamin B6 (mg/d) | A | <1.59 | 1.59-2.40 | ≥2.40 | <1.13 | 1.13-1.77 | ≥1.77 |
| Total folate (mcg/d) | A | <316.00 | 316.00-492.00 | ≥492.00 | <251.00 | 251.00-388.96 | ≥388.96 |
| Vitamin B12 (mcg/d) | A | <3.36 | 3.36-6.20 | ≥6.20 | <2.22 | 2.22-4.22 | ≥4.22 |
| Vitamin C (mg/d) | A | <42.44 | 42.44-113.21 | ≥113.21 | <38.01 | 38.01-98.49 | ≥98.49 |
| Vitamin E (ATE) (mg/d) | A | <5.82 | 5.82-9.42 | ≥9.42 | <4.53 | 4.53-7.52 | ≥7.52 |
| Calcium (mg/d) | A | <646.00 | 646.00-1072.00 | ≥1072.00 | <499.24 | 499.24-849.00 | ≥849.00 |
| Magnesium (mg/d) | A | <257.00 | 257.00-361.28 | ≥361.28 | <187.00 | 187.00-283.43 | ≥283.43 |
| Zinc (mg/d) | A | <9.75 | 9.75-15.10 | ≥15.10 | <6.73 | 6.73-10.75 | ≥10.75 |
| Copper (mg/d) | A | <1.12 | 1.12-1.57 | ≥1.57 | <0.85 | 0.85-1.28 | ≥1.28 |
| Selenium (mcg/d) | A | <94.94 | 94.94-141.80 | ≥141.80 | <67.79 | 67.79-99.50 | ≥99.50 |
| Total fat (g/d) | P | ≥69.83 | 69.83-107.43 | <107.43 | ≥50.98 | 50.98-75.79 | <75.79 |
| Iron (mg/d) | P | ≥12.88 | 12.88-19.17 | <19.17 | ≥9.65 | 9.65-14.32 | <14.32 |
| **Lifestyle OBS components** |  |  |  |  |  |  |  |
| Physical activity (MET-minute/week) | A | <417.86 | 417.86-1135.71 | ≥1135.71 | <270.00 | 270.00-845.71 | ≥845.71 |
| Alcohol (g/d) | P | ≥30 | 0-30 | None | ≥15 | 0-15 | None |
| Body mass index (kg/m2) | P | ≥25.54 | 25.54-29.17 | <29.17 | ≥23.74 | 23.74-28.64 | <28.64 |
| Cotinine (ng/mL) | P | ≥0.038 | 0.038-1.13 | <1.13 | ≥0.035 | 0.035-0.172 | <0.172 |

Abbreviations: OBS: oxidative balance score; A: antioxidant; P: prooxidant; RE: retinol equivalent; ATE: alpha-tocopherol equivalent; MET: metabolic equivalent

Table S2. Association of Metformin Use with All-Cause Mortality and Cardiometabolic Outcomes Among US Cancer Survivors by Age, Gender, BMI, and Race, NHANES 2003 to 2018

| **Treatment** | Metformin nonuser | Metformin user | | | | | |
| --- | --- | --- | --- | --- | --- | --- | --- |
|  |  | MV-adjusted HR (95% CI) ^a^ | | MV-adjusted OR (95% CI) ^b^ | | | |
| Subgroups |  | All-cause mortality | Cardiometabolic mortality | Total CVD | Stroke | Hypertension | CHD |
| **Age** |  |  |  |  |  |  |  |
| Elderly | 1 [Reference] | 0.62(0.47,0.83) | 0.64(0.42,0.97) | 0.43(0.29,0.64) | 0.44(0.25,0.78) | 0.54(0.35, 0.83) | 0.42(0.21,0.81) |
| Non-elderly | 1 [Reference] | 0.41(0.11,1.48) | 0.17(0.03, 0.99) | 0.41(0.15,1.17) | 0.67(0.20,2.23) | 0.34(0.09, 1.32) | 0.54(0.09,3.26) |
| Pinteraction |  | 0.51 | 0.58 | <0.001 | 0.07 | 0.02 | 0.02 |
|  |  |  |  |  |  |  |  |
| **Gender** |  |  |  |  |  |  |  |
| Women | 1 [Reference] | 0.54(0.35,0.81) | 0.73(0.43,1.23) | 0.47(0.27,0.81) | 0.54(0.28,1.03) | 0.32(0.14, 0.75) | 0.28(0.11,0.69) |
| Men | 1 [Reference] | 0.71(0.48,1.05) | 0.57(0.33,0.99) | 0.37(0.22,0.61) | 0.39(0.18,0.86) | 0.26(0.11, 0.61) | 0.50(0.24,1.04) |
| P interaction |  | 0.32 | 0.48 | 0.66 | 0.18 | 0.46 | 0.62 |
|  |  |  |  |  |  |  |  |
| **BMI** |  |  |  |  |  |  |  |
| Normal | 1 [Reference] | 0.66(0.36,1.23) | 0.99( 0.46,2.11) | 0.48(0.16,1.39) | 0.76(0.20,2.86) | 0.27(0.08,0.98) | 0.39(0.11,1.36) |
| Overweight | 1 [Reference] | 0.67(0.39,1.12) | 0.47( 0.23,0.98) | 0.64(0.31,1.32) | 0.51(0.21,1.25) | 0.34( 0.11,1.04) | 0.63(0.22,1.82) |
| Obese | 1 [Reference] | 0.63(0.42,0.94) | 0.55(0.31,0.99) | 0.33(0.19,0.57) | 0.32(0.16,0.66) | 0.22(0.09,0.51) | 0.34(0.15,0.81) |
| P interaction |  | 0.6 | 0.07 | 0.55 | 0.33 | 0.78 | 0.91 |
|  |  |  |  |  |  |  |  |
| **Race** |  |  |  |  |  |  |  |
| White | 1 [Reference] | 0.66(0.48,0.90) | 0.62( 0.39,0.98) | 0.39(0.25,0.61) | 0.40(0.21,0.76) | 0.23(0.10,0.54) | 0.41(0.20,0.87) |
| Black | 1 [Reference] | 0.58(0.35,0.96) | 0.47(0.21,1.08) | 0.41(0.19,0.89) | 0.66(0.25,1.73) | 0.71( 0.22,2.35) | 0.14(0.04,0.58) |
| Other | 1 [Reference] | 0.26(0.12,0.57) | 0.45(0.12,1.65) | 0.76(0.24,2.36) | 0.76(0.18,3.17) | 0.42( 0.10,1.70) | 0.47(0.16,1.42) |
| P interaction |  | 0.30 | 0.95 | 0.83 | 0.97 | 0.63 | 0.24 |

^a^ Multivariable adjusted Cox proportional hazards regression models: Adjusted for age, gender, race/ethnicity, educational level, family poverty income ratio, BMI, smoking status, alcohol use, physical activity, hyperlipidemia, diabetes, depression, antihyperlipidemic drug use, antihypertensive drug use, and years since the first cancer diagnosis.

^b^ Multivariable adjusted Logistic regression models: Adjusted for age, gender, race/ethnicity, educational level, family poverty income ratio, BMI, smoking status, alcohol use, physical activity, hyperlipidemia, diabetes, depression, antihyperlipidemic drug use, antihypertensive drug use, and years since the first cancer diagnosis.

Abbreviations: BMI, body mass index (defined as weight in kilograms divided by height in meters squared); MV, multivariable-adjusted; HR, Hazard Ratio; OR, Odds Ratio; CI, Confidence interval; CVD, Cardiovascular disease; CHD, Coronary heart disease; NHANES, the National Health and Nutrition Examination Survey.

Table S3. Association of Metformin Use with All-Cause and Cardiometabolic Mortality Risk Among US Cancer Survivors, NHANES 2003 to 2018

| Mortality Outcome | Hazard Ratio(95% CI) | | | |
| --- | --- | --- | --- | --- |
|  | Minimally adjusted model ^a^ | P value | Fully adjusted model ^b^ | P value |
| **All-cause mortality** |  |  |  |  |
| Treatment group |  |  |  |  |
| Metformin nonuser | 1 [Reference] |  | 1 [Reference] |  |
| Metformin user | 0.65(0.47, 0.89) | 0.007 | 0.66(0.50, 0.88) | 0.005 |
|  |  |  |  |  |
| **Cardiometabolic mortality** |  |  |  |  |
| Treatment group |  |  |  |  |
| Metformin nonuser | 1 [Reference] |  | 1 [Reference] |  |
| Metformin user | 0.65( 0.42, 0.99) | 0.043 | 0.65(0.43 0.97) | 0.037 |

^a^ Minimally adjusted model: Adjusted for age, gender, race/ethnicity, educational level.

^b^ Fully adjusted model: Further adjusted for family poverty income ratio, BMI, smoking status, alcohol use, physical activity, hyperlipidemia, diabetes, depression, antihyperlipidemic drug use, antihypertensive drug use, and years since the first cancer diagnosis.

Abbreviations: BMI, body mass index (defined as weight in kilograms divided by height in meters squared); CI, Confidence interval; NHANES, the National Health and Nutrition Examination Survey.

Table S4. Relative excess risk of all-cause mortality, cardiometabolic mortality due to antagonistic interaction effect of metformin use and oxidative stress levels in cancer survivors

|  | **All-cause mortality** | | **Cardiometabolic mortality** | |
| --- | --- | --- | --- | --- |
|  | **Death/No.** | **HR(95%CI)^a^** | **Event/No.** | **HR(95%CI)** **^a^** |
| Metformin user with low OS | 55/199 | 1.00(ref) | 31/199 | 1.00(ref) |
| Metformin nonuser with low OS | 437/1706 | 1.27(0.86, 1.89) | 165/1706 | 1.21(0.70, 2.11) |
| Metformin user with high OS | 65/240 | 0.92(0.55, 1.54) | 35/240 | 1.01(0.55, 1.84) |
| Metformin nonuser with high OS | 542/1716 | 1.61(1.08, 2.39) | 205/1716 | 1.68(1.03, 2.75) |
| **RERI** |  | **0.42(0.15, 0.69)** |  | **0.46(0.12,0.80)** |

^a^ Multivariable Cox proportional-hazards models were adjusted for age, gender, race/ethnicity, educational level, family poverty income ratio, BMI, smoking status, alcohol use, physical activity, hyperlipidemia, diabetes, depression, antihyperlipidemic drug use, antihypertensive drug use, and years since the first cancer diagnosis.

Abbreviations: HR, Hazard Ratio; CI, Confidence interval; RERI, Excess risk due to interaction; OS, Oxidative stress; BMI, body mass index.

Table S5. Association of Sulfonylurea Use with All-Cause and Cardiometabolic Mortality Risk Among US Cancer Survivors, NHANES 2003 to 2018

| Mortality Outcome | Hazard Ratio(95% CI) | | | |
| --- | --- | --- | --- | --- |
|  | Minimally adjusted model ^a^ | P value | Fully adjusted model ^b^ | P value |
| **All-cause mortality** |  |  |  |  |
| Treatment group |  |  |  |  |
| Sulfonylurea nonuser | 1 [Reference] |  | 1 [Reference] |  |
| Sulfonylurea user | 1.16(0.88, 1.53) | 0.30 | 1.15(0.87, 1.51) | 0.33 |
|  |  |  |  |  |
| **Cardiometabolic mortality** |  |  |  |  |
| Treatment group |  |  |  |  |
| Sulfonylurea nonuser | 1 [Reference] |  | 1 [Reference] |  |
| Sulfonylurea user | 1.15( 0.81, 1.64) | 0.44 | 1.16(0.82, 1.63) | 0.41 |

^a^ Minimally adjusted model: Adjusted for age, gender, race/ethnicity, educational level.

^b^ Fully adjusted model: Further adjusted for family poverty income ratio, BMI, smoking status, alcohol use, physical activity, hyperlipidemia, diabetes, depression, antihyperlipidemic drug use, antihypertensive drug use, and years since the first cancer diagnosis.

Abbreviations: BMI, body mass index (defined as weight in kilograms divided by height in meters squared); CI, Confidence interval; NHANES, the National Health and Nutrition Examination Survey.

Table S6. Correlations of Sulfonylurea Use with Four Specific Cardiometabolic Diseases Risk Among US Cancer Survivors, NHANES 2003 to 2018

| Cardiometabolic Comorbidities | Odds Ratio(95% CI) | | | |
| --- | --- | --- | --- | --- |
|  | Minimally adjusted model ^a^ | P value | Fully adjusted model ^b^ | P value |
| **Total CVD** |  |  |  |  |
| Treatment group |  |  |  |  |
| Sulfonylurea nonuser | 1 [Reference] |  | 1 [Reference] |  |
| Sulfonylurea user | 0.95(0.65,1.39) | 0.79 | 0.82(0.55,1.23) | 0.34 |
|  |  |  |  |  |
| **Stroke** |  |  |  |  |
| Treatment group |  |  |  |  |
| Sulfonylurea nonuser | 1 [Reference] |  | 1 [Reference] |  |
| Sulfonylurea user | 0.79(0.44,1.41) | 0.42 | 0.75(0.42,1.32) | 0.31 |
|  |  |  |  |  |
| **Hypertension** |  |  |  |  |
| Treatment group |  |  |  |  |
| Sulfonylurea nonuser | 1 [Reference] |  | 1 [Reference] |  |
| Sulfonylurea user | 0.92(0.52,1.62) | 0.76 | 0.69(0.35, 1.36) | 0.28 |
|  |  |  |  |  |
| **Coronary heart disease** |  |  |  |  |
| Treatment group |  |  |  |  |
| Sulfonylurea nonuser | 1 [Reference] |  | 1 [Reference] |  |
| Sulfonylurea user | 0.91(0.52, 1.58) | 0.73 | 0.73(0.41,1.31) | 0.29 |

^a^ Minimally adjusted model: Adjusted for age, gender, race/ethnicity, educational level.

^b^ Fully adjusted model: Further adjusted for family poverty income ratio, BMI, smoking status, alcohol use, physical activity, hyperlipidemia, diabetes, depression, antihyperlipidemic drug use, antihypertensive drug use, and years since the first cancer diagnosis.

Abbreviations: BMI, body mass index (defined as weight in kilograms divided by height in meters squared); CI, Confidence interval; NHANES, the National Health and Nutrition Examination Survey.

Table S7. Association between Metformin Use and All-Cause/Cardiometabolic Mortality Risk with further adjustment of HbA1c, Diabetic Retinopathy, GLP-1 Receptor Agonists Use and SGLT-2 Inhibitors Use

| Mortality Comorbidities | Multivariable adjusted HR (95% CI) ^a^ | P value |
| --- | --- | --- |
|  |  |  |
| **All-cause mortality** |  |  |
| Treatment group |  |  |
| Metformin nonuser | 1 [Reference] |  |
| Metformin user | 0.62(0.47, 0.82) | <0.001 |
|  |  |  |
| **Cardiometabolic mortality** |  |  |
| Treatment group |  |  |
| Metformin nonuser | 1 [Reference] |  |
| Metformin user | 0.65(0.43, 0.98) | 0.04 |

^a^ Multivariable adjusted Cox proportional hazards regression models: Adjusted for age, gender, race/ethnicity, educational level, family poverty income ratio, BMI, smoking status, alcohol use, physical activity, hyperlipidemia, diabetes, depression, antihyperlipidemic drug use, antihypertensive drug use, and years since the first cancer diagnosis, HbA1c, Diabetic Retinopathy, GLP-1 Receptor Agonists Use and SGLT-2 Inhibitors Use

Abbreviations: BMI, body mass index (defined as weight in kilograms divided by height in meters squared); CI, Confidence interval; HbA1c, Hemoglobin A1c; GLP-1, glucagon-like peptide-1; SGLT-2, sodium-glucose co-transporter 2.

Table S8. Correlations between Metformin Use and Four Specific Cardiometabolic Diseases Risk with further adjustment of HbA1c, Diabetic Retinopathy, GLP-1 Receptor Agonists Use and SGLT-2 Inhibitors Use

| Cardiometabolic Comorbidities | Multivariable adjusted OR (95% CI) ^a^ | P value |
| --- | --- | --- |
|  |  |  |
| **Total CVD** |  |  |
| Treatment group |  |  |
| Metformin nonuser | 1 [Reference] |  |
| Metformin user | 0.42(0.29,0.60) | <0.001 |
|  |  |  |
| **Stroke** |  |  |
| Treatment group |  |  |
| Metformin nonuser | 1 [Reference] |  |
| Metformin user | 0.48(0.29,0.81) | 0.01 |
|  |  |  |
| **Hypertension** |  |  |
| Treatment group |  |  |
| Metformin nonuser | 1 [Reference] |  |
| Metformin user | 0.29(0.15, 0.55) | <0.001 |
|  |  |  |
| **Coronary heart disease** |  |  |
| Treatment group |  |  |
| Metformin nonuser | 1 [Reference] |  |
| Metformin user | 0.42(0.22,0.81) | 0.01 |

^a^ Multivariable adjusted Logistic regression models: Adjusted for age, gender, race/ethnicity, educational level, family poverty income ratio, BMI, smoking status, alcohol use, physical activity, hyperlipidemia, diabetes, depression, antihyperlipidemic drug use, antihypertensive drug use, and years since the first cancer diagnosis, HbA1c, Diabetic Retinopathy, GLP-1 Receptor Agonists Use and SGLT-2 Inhibitors Use

Abbreviations: BMI, body mass index (defined as weight in kilograms divided by height in meters squared); CI, Confidence interval; HbA1c, Hemoglobin A1c; GLP-1, glucagon-like peptide-1; SGLT-2, sodium-glucose co-transporter 2.

Table S9. Association between Metformin Use and All-Cause/Cardiometabolic Mortality Risk Among US Cancer Survivors after Excluding Patients Receiving Dialysis in Past 12 Months, NHANES 2003 to 2018

| Mortality Comorbidities | Hazard Ratio(95% CI) | | | |
| --- | --- | --- | --- | --- |
|  | Minimally adjusted model a | P value | Fully adjusted model b | P value |
| **All-cause mortality** |  |  |  |  |
| Treatment group |  |  |  |  |
| Metformin nonuser | 1 [Reference] |  | 1 [Reference] |  |
| Metformin user | 0.67(0.49, 0.92) | 0.01 | 0.69(0.52, 0.91) | 0.01 |
|  |  |  |  |  |
| **Cardiometabolic mortality** |  |  |  |  |
| Treatment group |  |  |  |  |
| Metformin nonuser | 1 [Reference] |  | 1 [Reference] |  |
| Metformin user | 0.69( 0.45, 1.05) | 0.08 | 0.64(0.42, 0.97) | 0.04 |

^a^ Minimally adjusted model: Adjusted for age, gender, race/ethnicity, educational level.

^b^ Fully adjusted model: Further adjusted for family poverty income ratio, BMI, smoking status, alcohol use, physical activity, hyperlipidemia, diabetes, depression, antihyperlipidemic drug use, antihypertensive drug use, and years since the first cancer diagnosis.

Abbreviations: BMI, body mass index (defined as weight in kilograms divided by height in meters squared); CI, Confidence interval; NHANES, the National Health and Nutrition Examination Survey.

Table S10. Correlations between Metformin Use and Four Specific Cardiometabolic Diseases Risk Among US Cancer Survivors after Excluding Patients Receiving Dialysis in Past 12 Months, NHANES 2003 to 2018

| Cardiometabolic Comorbidities | Odds Ratio(95% CI) | | | |
| --- | --- | --- | --- | --- |
|  | Minimally adjusted model a | P value | Fully adjusted model b | P value |
| **Total CVD** |  |  |  |  |
| Treatment group |  |  |  |  |
| Metformin nonuser | 1 [Reference] |  | 1 [Reference] |  |
| Metformin user | 0.50(0.34,0.72) | <0.001 | 0.42(0.29,0.62) | <0.001 |
|  |  |  |  |  |
| **Stroke** |  |  |  |  |
| Treatment group |  |  |  |  |
| Metformin nonuser | 1 [Reference] |  | 1 [Reference] |  |
| Metformin user | 0.46(0.27,0.78) | 0.004 | 0.44(0.26,0.73) | 0.002 |
|  |  |  |  |  |
| **Hypertension** |  |  |  |  |
| Treatment group |  |  |  |  |
| Metformin nonuser | 1 [Reference] |  | 1 [Reference] |  |
| Metformin user | 0.49(0.27,0.89) | 0.02 | 0.26(0.14, 0.52) | <0.001 |
|  |  |  |  |  |
| **Coronary heart disease** |  |  |  |  |
| Treatment group |  |  |  |  |
| Metformin nonuser | 1 [Reference] |  | 1 [Reference] |  |
| Metformin user | 0.55(0.28, 1.06) | 0.08 | 0.46(0.23,0.92) | 0.03 |

^a^ Minimally adjusted model: Adjusted for age, gender, race/ethnicity, educational level.

^b^ Fully adjusted model: Further adjusted for family poverty income ratio, BMI, smoking status, alcohol use, physical activity, hyperlipidemia, diabetes, depression, antihyperlipidemic drug use, antihypertensive drug use, and years since the first cancer diagnosis.

Abbreviations: BMI, body mass index (defined as weight in kilograms divided by height in meters squared); CI, Confidence interval; NHANES, the National Health and Nutrition Examination Survey.
